# Importance score of SARS-CoV-2 genome predicts the death risk of COVID-19

**DOI:** 10.1038/s41420-022-01100-7

**Published:** 2022-07-02

**Authors:** Chunmei Cui, Qinghua Cui

**Affiliations:** Department of Biomedical Informatics, MOE Key Lab of Cardiovascular Sciences, School of Basic Medical Sciences, Peking University, 38 Xueyuan Rd, 100191 Beijing, China

**Keywords:** Viral infection, Outcomes research

The death risk of COVID-19 caused by SARS-CoV-2 seems strikingly decreased due to extensive vaccination and attenuated pathogenicity of SARS-CoV-2 variants [1–3], however, we still face big challenges in monitoring the long term trend and warning the death risk of COVID-19 because the epidemiological situation is continuously evolving as the pandemic has entered the third year [4]. We previously presented efficient algorithms [5, 6] to quantify the importance score of both coding RNA molecules (mRNAs) and noncoding RNA molecules (long noncoding RNAs and microRNAs) based on sequences. Given that SARS-CoV-2 is a single-stranded positive-sense RNA virus, here we used the GIC (Gene Importance Calculator) algorithm to compute the importance score (GIC score) of 1,019,300 SARS-CoV-2 complete genome RNA sequences collected between December 2019 and now (May 2022) from the NCBI SARS-CoV-2 database (https://www.ncbi.nlm.nih.gov/sars-cov-2/). The GIC algorithm was trained with a logistic regression model based on nucleotide triplet features and secondary structure info derived from RNA sequences to evaluate the importance of RNAs. Subsequently, to quantify the death risk of COVID-19, we obtained the reported counts of cases and deaths from WHO Coronavirus (COVID-19) Dashboard (https://covid19.who.int/info). And then we estimated the monthly death risk by calculating the death-to-case ratio for every month. For exploring the association between genome RNA sequences of SARS-CoV-2 strains and their death risk, we calculated the correlation of GIC score of virus RNA sequences and death risk of COVID-19 with Spearman’s correlation analysis. Interestingly, we found that the monthly death risk of COVID-19 is highly related with the importance score of the genome RNA sequences of SARS-CoV-2 strains (Fig. 1). Moreover, February 2021 shows to be an inflection point for their relation. Before February 2021, the death risk of COVID-19 has highly negative correlation with the GIC score (Rho = −0.86, p-value = 8.20E−5; the left part of the dotted line in Fig. 1a), whereas their relation clearly turns into positive correlation (Rho = 0.75, p-value = 1.18E−3; the right part of the dotted line in Fig. 1a) and the death risk decreased obviously after February 2021. Strikingly, we revealed that it is just before February 2021 the number of countries beginning with the first vaccine increased sharply (44 countries on December, 2020 and 34 countries on January, 2021) according to the WHO data (Fig. 1b), suggesting that vaccination generated a great effect on both the genome and the severity of the virus. Meanwhile, the number of types of SARS-CoV-2 lineages and sublineages significantly decreased after the prevalence of vaccination, that is, since January 2021 (Fig. 1c), while virus still could evolve into new variants and produce further threat to public health, like Omicron variant (BA.1, BA.2, BA.3, and BA.4). The above findings suggest that the virus would produce serious effects on health again once current vaccines fail to protect against new variants in the future. Finally, we developed an online tool CoVIS (https://www.cuilab.cn/cov2) for timely calculating GIC score and thus monitoring the tendency of death risk of COVID-19.

**Fig. 1:**
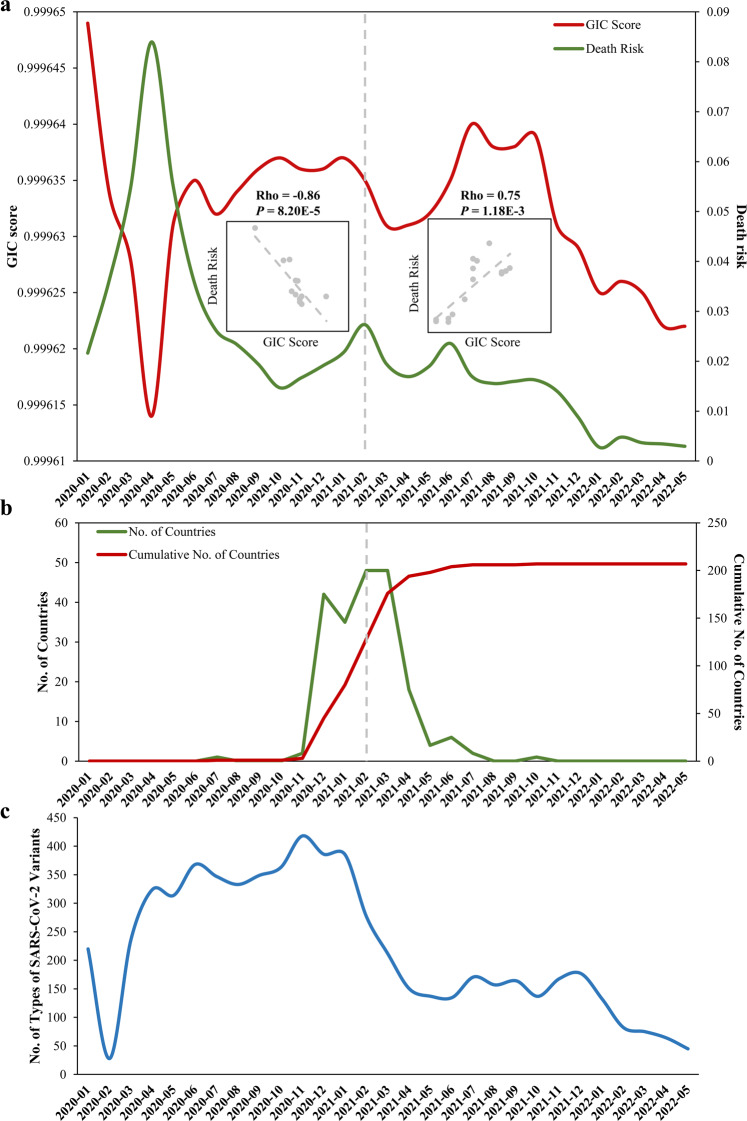
Monthly changes of the importance (GIC) score of SARS-CoV-2, the death risk of COVID-19 caused by the virus, and the number of countries beginning with the first vaccine. **a** shows that relation between the GIC score and the death risk. Their relation is negatively and positively correlated before and after February 2021, respectively. **b** shows the number and cumulative number of countries beginning with the first vaccine. **c** shows the number of types of SARS-CoV-2 variants.

## Data Availability

Data are available to the journal and the publisher upon request.
